# Evidential Incognizance

**DOI:** 10.1007/s12136-024-00608-0

**Published:** 2024-09-25

**Authors:** Simon Rippon

**Affiliations:** Department of Philosophy and Department of Public Policy, Central European University (CEU), Quellenstrasse 51, 1100 Vienna, Austria

**Keywords:** Epistemic vice, Propaganda, Epistemology of democracy, Evidence, Sensibility

## Abstract

In this article, I explore an epistemic vice I call “evidential incognizance.” It is a vice of failing generally to recognize evidence, or recognize the full force of evidence, in a domain of knowledge. It frequently manifests as a kind of unbridled skepticism or hopelessness about knowing in the domain, including (but not limited to) skepticism about expert testimony. It is epistemically vicious primarily because it leads people to overlook valuable epistemic opportunities, and thus tends to obstruct knowledge and justified belief. I believe it is of interest particularly because it tends to arise as a reaction to a certain kind of information environment and is often induced intentionally by populist candidates and authoritarian regimes. I discuss the nature of evidential incognizance, its relation to and differences from other epistemic shortcomings, its political significance, why it may have been previously overlooked in the literature, and the potential for overcoming it.

## Introduction

While it is arguable that not all justified belief and knowledge depends on evidence, plenty of it surely does. A detective cannot know who committed a murder without evidence, you cannot know what time the bus will come without evidence, and voters cannot have justified beliefs about which party they should vote for without evidence.^1^ The last example highlights an essential role that evidence plays in democracies. One might assume that voters would typically need evidence about—*inter alia*—alternative candidates, parties and platforms, offices and institutions and their respective powers and responsibilities, the historical performance of officials and candidates, and the prevailing social and economic conditions, in order to know who to vote for. But this article is not about the quantity, quality, or sources of evidence that voters need. It rather concerns their ability to avail themselves of evidence that exists. For if anyone is to know or to form justified beliefs on the basis of evidence, they must necessarily recognize the evidence (implicitly or explicitly) *as* evidence of at least some minimal value. If epistemic vices are objectionable aspects of character that tend to inhibit the production, retention, and transmission of justified belief and knowledge, then it follows that some epistemic vices could obstruct knowledge by obstructing our recognition of evidence.^2^ A fully epistemically virtuous person, among other things, would be disposed to perceive and be sensitive to the evidence available to them in such a way that each piece of evidence is allocated due weight in the process of belief formation and revision.

In this article, I focus on a common but previously unremarked epistemic vice that I call “evidential incognizance.” This vice consists in a kind of perceptual insensitivity or incognizance, in which one either fails to recognize evidence at all (when one should recognize it), or credits the evidence diminished value, for broad swathes of the evidence in a specific domain of knowledge. I suggest that evidential incognizance plays an important role in politics, producing a widespread kind of unbridled skepticism or apathy among voters, and this turns out to be useful to politicians of a certain stripe. I will argue further that because it is beneficial to them, such politicians have learned to bring about conditions that breed evidential incognizance. And finally, I will suggest that philosophy not only helps us to understand this vice, as I hope it does here, but may well assist us in overcoming it.

## Evidential Incognizance—A Portrait

In this section, I describe the nature of evidential incognizance and relate it to some other kinds of epistemic failing. I start with a model on which knowing rests on an initial perception of evidence. This may sound like a strong assumption to make, but two qualifiers mitigate its strength: first, the perception in question may be implicit rather than explicit (one might come to explicitly recognize one’s evidence for what one knows only when asked a question such as: “How do you know that?”, or one might never come to explicitly recognize it at all); second, I do not claim that it is true of *all* knowing that it rests on perception of evidence, but only of plenty of it. On this model, then, our perception of evidence for propositions is often a precondition for our knowing them. This should not be controversial. Even a reliabilist externalist, for example, should accept this claim, since the objective fact of our reliability in many cases depends upon our perception of evidence.

The fact that knowledge often depends on perception of evidence is important, because the perception of evidence involves the exercise of capacities; capacities can vary according to natural talent or acquired skill; and exercises of capacities depend upon motivation. So here, the language of epistemic virtue and vice can be brought to bear. We might think of the relevant virtue or collection of virtues here as those which tend to bring about a due appreciation of evidence. So, the correlate vices would be those that tend to lead to either an under-appreciation or an over-estimation of (parts of) the evidence.

One well-known way that we can under-appreciate evidence is by lacking motivation to care about it. Lack of curiosity (lack of desire to inquire or to know), closed-mindedness (excessive desire for firm answers), dogmatism (excessive attachment to a particular doctrine), or wishful thinking (misdirected desire for a particular claim to be true) may motivate us to overlook evidence, or reduce our motivation to seek it, particularly when the evidence is inconvenient. But evidential incognizance, the vice I describe here, is not to be understood in the first instance as a motivational failure (though it does tend to undermine motivation, more on that later). It is better described as a kind of induced failure to be able to see evidence, a non-wilful blindness to evidence. Or more accurately, it is a kind of induced dullness to evidence *as* evidence, or to its value *as* evidence. It is as if one’s evidential “antennae” were dimmed or turned off. If we cannot see the evidential import of what confronts us in the first place, then even with all the motivation in the world, we could not respond to it in an epistemically appropriate way.

There are different kinds of blindness to evidence that are non-vicious, and I want to distinguish these from evidential incognizance. The first is the sort of case in which the development of special expertise may furnish either a background theory or refined perceptual capacities that enable an expert to glean evidence that a lay person would normally be insensitive to.^3^ What, for example, to an ornithologist might present itself as strong evidence of the presence of a tufted titmouse, might be entirely overlooked or recognized only as signs of the presence of some-bird-or-other by an average person (even if they just happened at that moment to be unusually interested in the question of which bird species were present)! We should not regard most people as epistemically vicious for failing to have developed the ornithologist’s expertise. There may be cases in which evidential incognizance manifests in failing to develop a background theory or perceptual capacities that, in contrast to ornithological expertise, ordinary people *should* develop. But I suspect it is more usually manifest in a dulling of capacities that are or were previously possessed.

A second kind of non-vicious blindness to evidence that I want to distinguish from evidential incognizance is blindness to evidence pertaining to matters that are not deserving of one’s attention.^4^ Curiosity is not always a virtue: one can certainly be excessively curious about things that are none of one’s business. Moreover, one is surely not obliged to form beliefs about everything one happens to have access to evidence about. Here are two examples: First, the fact that one *could* count the number of leaves on a leafy tree does not make it the case that one *ought to* do so (nor that one ought to form a—useless and uninteresting—true belief about how many leaves there are). Second, any evidence one encounters for some proposition *that p* is *ipso facto* also evidence for the disjunctive proposition, for any arbitrary *q*, *that p or q*. But one cannot be considered vicious for failing to see all one’s evidence at the same time as evidence for an infinite range of arbitrary disjunctive beliefs, and for failing to form these (irrelevant and uninteresting) disjunctive beliefs accordingly. Our concern here is rather with blindness to evidence that one *should be* attentive to; evidence for propositions that *matter*. The examples at the outset of this article were platitudinous examples of evidence for propositions that matter.

A different phenomenon to distinguish from evidential incognizance in my sense is the kind of inattention that would oppose the virtue of being *observant*, in the sense defined by Christopher Hookway (2003a, 2003b, 2006). Hookway distinguishes three levels at which excellence is necessary for the epistemically virtuous inquirer: First, a basic excellent perceptual skill or capacity such as good eyesight; second, a trait of affectively tuned sensitivity to the information perceptually available, reflecting background knowledge and expectations, such that we tend to observe the facts we (ought to) regard as relevant to our epistemic goals and needs. This is what Hookway characterizes, in the case of vision at least, as *being observant* (Hookway, 2006, p. 67). And third, awareness of the relevance of the facts we observe to our cognitive goals, so that we can make good use of the acquired information and attend appropriately, managing our trait of being observant well.

The vice of evidential incognizance—that of tending to fail to recognize evidence in some domain *as* evidence of due weight—is strictly compatible with being observant in Hookway’s sense, since it is consistent with a tendency to observe all the salient facts (and indeed evidential incognizance is consistent with noticing all the right facts *as* salient, if not for the right reasons). That said, no one who is evidentially incognizant will be fully epistemically virtuous, since even if they are observant, they will fail to recognize the (full) evidential force of what they observe. Furthermore, acquiring evidential incognizance in a domain would almost certainly tend to disrupt one’s affectively tuned sensitivity to relevant information within that domain, undermining one’s ability to be observant. Thus, evidential incognizance and the vice of inattention that opposes Hookway’s virtue of being observant are closely related, though distinct.

There’s another influential view in the literature that bears a relation worth exploring to the current inquiry. Like my proposal, it adopts a perceptual epistemic model and identifies a vice of under-appreciation of evidence. I have in mind Miranda Fricker’s model of social credibility judgment, as related in her book *Epistemic Injustice* (Fricker, 2007). According to Fricker, in cases of what she calls “testimonial injustice,” the hearer’s identity prejudice “distorts the hearer’s perception of the speaker,” raising doubts about competence or sincerity and causing him to unjustifiably view her as untrustworthy (2007, p. 36). On the other hand, “training instils in the virtuous hearer empirically well-grounded habits of epistemically charged social perception, and thus reliable perceptual judgements of speaker credibility” (2007, p. 5).

Speaker credibility is a perfectly good focus for Fricker, given her primary interest in understanding injustices we do each other as communicative agents. But we can straightforwardly broaden the subject to evidential value more generally. In cases of testimony, it so happens that when *p* is asserted, the evidential value for the proposition that *p* of the fact of its being said corresponds to the credibility of the speaker in saying *p*. If our interest is in understanding epistemic vices in the context of a perceptual model in epistemology, we should broaden our focus beyond testimony. After all, it makes little sense to limit a perceptual model to credibility judgments, rather than to suppose that we more generally perceive evidence, including testimony but also other kinds of evidence, as having evidential value. My more general proposal here—a perceptual model of evidential value—would be a much closer epistemic analogue to the models of moral perception developed by authors like David McNaughton (1988) in metaethics. Indeed, the theorist probably most responsible for inspiring McNaughton’s views in metaethics, John McDowell, explicitly extends the idea of perception of specifically ethical reasons to the realm of reasons in general:

[H]uman beings are intelligibly initiated into this stretch of the space of reasons by ethical upbringing, which instils the appropriate shape into their lives. The resulting habits of thought and action are second nature … The point is clearly not restricted to ethics. Moulding ethical character, which includes imposing a specific shape on the practical intellect, is a particular case of a general phenomenon; initiation into conceptual capacities, which include responsiveness to other rational demands besides those of ethics… If we generalize the way Aristotle conceives the moulding of ethical character, we arrive at the notion of having one's eyes opened to reasons at large by acquiring a second nature. (McDowell, 1996, p. 84).

McDowell holds, then, that the intrinsically conceptualized nature of mature human perception that is provided by an ordinary, decent upbringing provides us with perceptual sensitivity to reasons in general. While McDowell’s own interests are primarily to stake out a position in the realism/anti-realism debate and respond to Sellars’ problem of the “myth of the given,” we need not follow his broader program to accept the perceptual model of evidential value that I adopt here.

Another concept that has cropped up from time to time in the epistemic vice literature that should be distinguished from that of evidential incognizance is that of “myopia.” Ian James Kidd helpfully identifies myopia as “an umbrella term for a set of epistemic failings,” as “a *stance*, a structure of assumptions, attitudes, beliefs, and character traits” which involves, in opposition to the epistemic values of broad-mindedness and depth, having a narrow or shallow vision of something (Kidd, forthcoming). Given that there are many reasons one can develop a narrow or shallow vision of things, and many different ways in which one’s vision can be narrow or shallow, I agree that it makes sense to treat the concept of myopia as an umbrella term. But much of what Kidd has in mind is clearly very closely related to being closed-minded or dogmatic in terms of the framework within which one views an area of inquiry. Kidd writes, “If I am myopic about P, I am more susceptible to caricaturing it. If I have a myopic understanding of a situation, I will fail to discern complexities and problems in ways which incline to me arrogant overconfidence.” If that is what is meant by “myopia,” then it is quite different from what I have in mind when I talk about evidential incognizance, since the latter is more closely related to under- than to overconfidence. Myopia, or short sight, allows some (near) things to remain in sharp focus while it blurs out others. If we want a visual metaphor for evidential incognizance, perhaps a better metaphor would be a generalized dimming of vision.

Aristotle thought that every ethical virtue is a mean between excess and deficiency (350 B.C.E./2009 1106a26–b28). Arguably, this doctrine of the mean could be extended also to many epistemic virtues (cf. Crisp, 2010; King, 2021). So if evidential incognizance is an epistemic vice of deficiency of assessment of epistemic value, we can ask what might be its Aristotelian opposite vice? That would be a vice of generally perceiving things one encounters in a particular domain of knowledge as having excessive, exaggerated evidential value. It seems likely that someone with such an amplified view of evidence could be disposed toward pseudoscientific thinking, in which case vice terms such as credulity or confirmation bias may apply.^5^ On the other hand, if we suppose that the individual’s tendency to hallucinate or over-estimate evidence in the domain is really generalized, one can imagine the person as reduced to a paralysis of indecision as they encounter apparent “counter-evidence” for every hypothesis! So perhaps paralysis is the opposite vice. Perhaps we should say that evidential incognizance has not one but two opposite vices: credulity and paralysis; or perhaps we should prefer to say that the doctrine of the mean does not straightforwardly apply here.

At any rate, my core thesis is this: *knowledge is often grounded in perception of evidential value, and a generalised dulling of perception of evidential value with respect to broad swathes of evidence in a domain of knowledge is the epistemic vice I call evidential incognizance*.

## Production of Evidential Incognizance

My own interest in evidential incognizance arose from interests in propaganda and in the epistemology of democracy, and in particular interest in what can go wrong epistemically in backsliding or otherwise hollow democracies. I used to think of propaganda as usefully defined roughly this way:

- Propaganda = _def_ An attempt to persuade a mass audience in ways that circumvent or short-circuit critical faculties.

This fits a certain model of propaganda, which I’ll call the “old” model. In some times and places, governments had a near monopoly over what information citizens had access to. At the same time, state media could be used to present an official version of events which was designed to be coherent and persuasive, though it may have borne little resemblance to the truth.

In the age of widespread access to the internet and social media, while autocratic governments may ensure that they, together with their allies, dominate mass media through financial and regulatory means (as they do, for example, in today’s Hungary or India), and while some governments still combine this kind of domination of mass media with significant censorship activity (as in Russia), the idea of a government—even less a political campaign—possessing anything approaching an information monopoly has become untenable. This is at least true of all countries except those which exercise repressive, systematic controls over access to information on the internet (think of North Korea or perhaps China) (RSF, 2023). Though internet access is by no means universal, in most states it is no longer possible to prevent much of the population having access to ideas and opinions which certain governments would like to repress. How has propaganda evolved to respond to this changing environment? In contrast to the “old” model, an interesting fact about contemporary propaganda is that much of it doesn’t seem to seriously attempt to persuade its audience of one version of events at all, but rather to sow more general doubt and confusion. I placed scare quotes around the “old” of “old model” here intentionally, since persuasion may never have been as central to, say, Soviet propaganda as one might assume (Pomerantsev, 2014).

One thing autocratic governments and political campaigners can do with respect to institutions, individuals, and ideas they cannot repress is seek to discredit them. This they certainly do, in myriad ways. But one observes that the character of these attempts to discredit is typically not so much targeted criticism, following a Millian liberal ideal of engagement with ideas and arguments in the public square, as sweeping dismissals designed to discredit large swathes of opposition and independent commentary all at once (Mill, 1859/1978).

For example, in recent years, the Hungarian Fidesz government led by Viktor Orbán has issued several so-called “National Consultations,” surveys issued to every household that are ostensibly intended to consult citizens on important policy matters, but are filled with loaded questions designed to elicit favored answers, to create the false impression of near-universal consensus on the government’s policies, and to caricature and demonize opponents (Batory & Svensson, 2019).

In addition, the Fidesz government has promoted a nationalist and populist “us” and “them” narrative. It has routinely dismissed analysis and criticisms of its policies and its declining democratic institutions by independent non-governmental organizations, EU bodies, independent media, and others, as products of the “Soros empire” or “foreign agents.” It has extensively used the state- and privately owned media, government advertising budgets, and even the law to portray its various opponents as enemies of Hungary and of traditional Hungarian families. Examples of the latter include, for example, the law on foreign-funded NGOs, which required organizations receiving any significant funding from abroad to label themselves on their websites and in all published materials as “organisations receiving foreign funding” (HCLU, 2017), and the “anti-LGBTQI Law,” Act LXXIX of 2021, which was written and promoted by ministers in a way that associated homosexuality with pedophilia (Priebus & Végh, 2022).

A similar populist “us” and “them” approach has been observed in other states including Russia (which, long before Hungary, passed a “foreign agents” law to brand and discredit independent NGOs) (Seskuria, 2021), as well as more broadly in political campaigns such as those of Donald Trump or the Vote Leave campaign in the UK’s 2016 Brexit referendum. Donald Trump has constantly attacked what he refers to as the “Fake news” media and the “deep state” and repeated the slogan “America First!” suggesting that only he is on the side of ordinary Americans (National Archives, 2019). The Vote Leave campaign, in dismissing forecasts and warnings about the consequences of leaving the EU, constantly attacked “Brussels bureaucrats” and a supposed out-of-touch elite who wanted to tell ordinary people what was best for them, sentiments encapsulated in Michael Gove’s infamous comment that “people in this country have had enough of experts” (Clarke & Newman, 2017). These populist strategies function to discredit and promote the wholesale dismissal of independent opinion and analysis as well as political opponents. If the government or the campaign speaks for “us,” then whatever resistance there is to it must be coming from some outside “them,” which makes it, first, an opinion it is less natural to identify with, and second, automatically suspect in virtue of the range of potentially hostile or self-serving motives the “others” propounding the alternative opinions may have. They serve as an invitation precisely *not* to listen and engage with the arguments, but to dismiss them.

Another range of “new” propaganda tactics that are designed not to persuade but to sow doubt, discord, fear, cynicism, and fatigue we can loosely describe as “noise tactics,” because they primarily serve to devalue the quality of information citizens are exposed to, rather than add anything intended to be perceived as a serious contribution to the social discourse. One example that will surely be familiar to all readers is trolling, especially on social media and in the comment sections on news websites. Trolls, including fake online personas known as “sock puppet” accounts (whose posts may be human- or bot-generated), are used to spread rumors and conspiracy theories, to silence journalists and others with abuse and harassment, to divide, mislead, or inject doubt about where public opinion stands, and to distract and confuse. One Russian “troll farm,” the Internet Research Agency (IRA), a company occupying an office block in St. Petersburg, infamously attempted to influence the US elections in 2016 in favor of Donald Trump as well as targeting other countries and, primarily, the Russian domestic audience (Dawson & Innes, 2019). There can be no doubt about the IRA’s ties to the Kremlin: Yevgheny Prigozhin, the oligarch and leader of the state-funded Wagner Group private military company who had a close relationship with Vladimir Putin until he instigated a rebellion in 2023, was its admitted founder (Hopkins, 2023).

Noise tactics, in this sense, extend far beyond social media. TV producer and writer Peter Pomerantsev provides vivid accounts of Russian information operations based partly on his own experience in the employ of state and private television channels (Pomerantsev, 2014, 2019). He observes that Russian television takes on the flashy graphics and trappings of western reality TV, news, and debate shows, but retains a “managed” diversity of opinion. The shows quench the audience’s appetite for debate and provide a façade of democracy, but platform only a puppet opposition that presents no threat to the Putin regime. Government-friendly views remain dominant in the mass media, but there’s no need for a coherent or consistent government line to be promoted; the story can change with bewildering speed from one day to the next (indeed, it sometimes seems intentional that it does so) (Pomerantsev, 2014). To give an example that will be familiar to most readers, one is reminded of Vladimir Putin’s varying explanations for his military invasion of Ukraine, from his first year of denial (there are no Russian soldiers in Crimea, only curiously taciturn “local self-defence units” who wear often uniforms without insignia similar to those of the Russia army), to “protection” of ethnic Russians from “genocide” and “denazification,” to a supposed NATO threat, to a historical claim to reunify the land. Pomerantsev’s picture of Russian media and politics is of a surreal place in which everything is instrumentalized (there is only “political technology” rather than political philosophy), nothing is real, and nobody really believes or believes *in* anything. His former television colleagues are untroubled: “‘Over the last twenty years we’ve lived through a communism we never believed in, democracy and defaults and mafia state and oligarchy, and we’ve realized they are illusions, that everything is PR.’ ‘Everything is PR’ has become the favourite phrase of the new Russia; my Moscow peers are filled with a sense that they are both cynical and enlightened,” he writes (Pomerantsev, 2014, p. 73). This “PR” does not stop at the boundaries of media and social media. Pomerantsev also writes about fake opposition politicians and interference in foreign countries, including supposedly "grassroots" Russian-friendly protests that are atually orchestrated by the Kremlin, with trappings that mimic prior opposition protests such as Maidan. He notes that all of these propaganda activities can be effective even when the Kremlin’s influence becomes apparent, since such discoveries promote the Kremlin narrative that *everywhere* democracy is hollow, that *all* social movements are secretly orchestrated by states, and that there is no such thing as objective media (Pomerantsev, 2019).

A *Financial Times* report of complaints about Russian conduct in the UN Security Council suggests awareness of the “new” propaganda strategy:

[Western officials] highlight how traditionally Russia never wanted to give civil society much access to Security Council debates but since its full-scale invasion of Ukraine has invited dozens of outside figures to speak about the war — including Roger Waters, the former Pink Floyd bassist, who accuses the west of provoking the invasion. ‘They are degrading the level of debate in the Security Council by inviting unqualified briefers and conspiracy theorists who make the debate look like a circus,’ said one western diplomat. ‘It makes an onlooker think it’s not worth listening to.’ (Russell & Schwartz, 2023).

The “new” propaganda, then, results not in a highly controlled but rather in a *noisy* epistemic environment, in the sense that it contains lots of unreliable information and distraction. At the same time, it sows doubt and undermines trust in institutions, in objectivity, and in independent analysis and expertise. My contention is that the onset of evidential incognizance is an entirely natural response to exposure to such an epistemic environment. The “new” propaganda is thus what Ian James Kidd (2019) calls an “epistemically corrupting” social influence, causing agents to develop the epistemic vice of evidential incognizance. Insofar as the propaganda is effective, voters come to see many of the best sources of evidence as not worth engaging with. Finding the signal of truth in a noisy, distracting, devalued epistemic environment is difficult and complex, and a sense of confusion and loss of ability to rationally form true beliefs—that is, of epistemic agency—would be a natural consequence of finding oneself in such a situation. It is then all too easy to give up on rational inquiry and to disclaim responsibility for knowing (“The war? It’s a question for the politicians!”), to lapse into a cynical unbridled skepticism (“Everything they say is rubbish!”), or to take a path of least resistance, following the crowd or one’s gut instincts. This, of course, perfectly serves those populist politicians who are willing to pander to our worst prejudices.

The present proposal bears an interesting analogy to a much-cited finding in psychology known as “learned helplessness.” In one of the original (disturbing!) experiments (Seligman & Maier, 1967), the first stage was to expose dogs trapped in harnesses to electric shocks that could be switched off by pressing a panel with their heads, while a second group of dogs trapped in harnesses was exposed to inescapable electric shocks of effectively random duration. The first group of dogs learned to press the panel to escape the shock while in the harness. In the second stage, these two groups of dogs plus a third, control group were put individually in a very different environment: a shuttle box (an enclosure with a mesh floor, divided by a low barrier). An electric shock was applied to one side of the enclosure, which the dog could easily escape by jumping over the low barrier. It was found that not only almost all dogs in the first group, who had learned to press the panel in the first stage of the experiment, but also those in the third, control group, would jump to escape the shock. However, most dogs in the second group, who had earlier been exposed to the uncontrollable shocks, did not even attempt to jump the barrier to escape. They had unlearned, or perhaps never learned, that they could exercise their agency to escape the trauma. The learned helplessness experiments have been widely replicated in other animals and humans, and the results have been used to support a theory of human clinical depression according to which it is a loss of agency explained by previous exposure to uncontrollable aversive events (Maier & Seligman, 2016). The analogy between these types of learned helplessness and evidential incognizance, then, would be that if someone is put into a chaotic, noisy epistemic environment in which they have a diminished ability to exercise their epistemic agency, they may then fail to see that they can exercise epistemic agency even when they are able to do so. And failing to see that one can exercise epistemic agency may consist just in having a dulled perception of evidence, that is, evidential incognizance.

For plausible examples of how evidential incognizance is induced beyond the purely political domain, consider the type of doubt-sowing engaged in by public relations firms and a few scientists from the 1960s on behalf of the tobacco industry, in relation to the link between smoking and cancer, and still now on behalf of the fossil fuel industry, in relation to climate change. Their efforts to manufacture the appearance of serious scientific doubts and controversy about well-established findings, together with a widespread tendency in the media to seek to present “both sides of the debate” on too many issues, not just in pursuit of ratings but often in misguided pursuit of objectivity, have often ended up presenting a false balance to the public, leading many people to fail to recognize the evidential value of scientific findings and credible sources in these domains.^6^

## Challenges

I have argued that there is an epistemic vice, evidential incognizance, which is constituted by dulling of perceived evidential value with respect to broad swathes of evidence in a domain of knowledge. I have argued that in the political domain, this vice can explain certain familiar kinds of widespread skepticism and apathy, and that it is targeted by the “new” model of propaganda.

One potential objection to my account is that if evidential incognizance is a genuine epistemic vice distinct from others, and if it plays such an important political role, how come no one has noticed it before? One potential explanation begins from the fact that the vice manifests what Jose Medina (2013, p. 75) calls meta-blindness: not only are those who have it blind to evidence, but they are also blind to the fact that they are blind to evidence. If you have evidential incognizance, you won’t know that you have it. Furthermore, evidential incognizance is what we might call a “modest” vice: unlike those who are arrogant or dogmatic, for example, those who have it aren’t likely to express themselves loudly and forcefully because they are simply not likely to think that they know very much, or know very firmly, about matters in the domain in question. Indeed, they may well develop a sense of hopelessness that generally leads them to give up trying to talk, or think, about matters in that domain. A second potential explanation is a social one, starting from the hypothesis that we can expect a negative correlation to hold between class or social dominance and evidential incognizance: if you are socially dominant, you are accustomed to being empowered, and therefore less likely to think that (epistemic) agency is beyond your grasp. Evidential incognizance may then have been overlooked because, to put the point bluntly, it primarily affects members of society we are not accustomed to listening to, or otherwise paying enough attention to.

A second objection may accuse me of “talking down” to voters I don’t agree with and accusing them of irrationality or, even worse, of vice. It may be argued that voters for populist candidates and authoritarian leaders are rationally expressing their strongest preferences, or legitimately responding to the consistent failure of mainstream politicians to improve their material conditions. I need not disagree with any reasonable version of these claims; I don’t claim that *all* those who vote for populists and authoritarians do so under the influence of evidential incognizance. Nor do I claim that those who *don’t* vote in these ways vote “rightly” or rationally or free from the influence of epistemic vices. It is hard to see how anyone could seriously deny that at least *some* voters are influenced by epistemic vices. Admittedly, I have suggested that some voters are influenced by an epistemic vice (and I hasten to clarify: this is no accusation of *moral* vice), but I have also emphasized that their having it is in large part the responsibility of those who debase the epistemic environment they inhabit.

Still, it wouldn’t do to accuse others of an epistemic vice without examining whether I might not be subject to the same. One area in which I suspect I might suffer evidential incognizance myself, though for very different reasons than those I canvassed in the political case, is in the domain of religion. If I were a convinced religious believer, my failure to see the force of evidence, and also to seek it, could easily be ascribed instead to the distinct epistemic vice of wishful thinking: my desire for an ordered universe and all-powerful, benevolent God might motivate me to ignore or underestimate the evidence against it. Perhaps a determined atheist could equally be motivated by wishful thinking, if he had a strong desire for the truth of scientific naturalism, say. But personally, I neither wish for the existence of God nor the absence of God. I would describe myself as an atheist leaning toward agnostic. I find myself pulled toward the view that it’s just not that evident which way to go on religious questions; I would say, if pressed, that religious belief is a matter of reasonable disagreement. I wonder, however, if this impression might not be due to evidential incognizance motivated by a desire to live comfortably and respectfully in a pluralistic society with friends of all religions and none. For if I decided it *were* entirely evident that there is no God (or indeed that there is one), wouldn’t I have to accept that many of my friends, and others I deeply admire, have been woefully obtuse about the matter? Perhaps the easy way out is to be blind to the force of the evidence in the domain so that we can just get along. To be clear, I don’t know that I suffer evidential incognizance in the religious domain (as I have said, evidential incognizance involves meta-blindness), but this is some reason for suspicion.

## Philosophy as a Corrective to Evidential Incognizance

This article has been primarily focused on identifying an under-appreciated epistemic vice and understanding its significance. But naturally, it raises the question: what shall we do about evidential incognizance? I don’t seek to offer a final answer to this question; in my view, there is a need for empirical work on the scope of the problem in practice and to explore the effectiveness of potential remedies. I will observe, though, that some examples of evidential incognizance I have suggested result either from an excessively broad skepticism—an over-generalization from “much such-and-such evidence is worthless” to “all such-and-such evidence is worthless” (this seems clearest in the typical political cases), or from a kind of inattention (this seems clearest in the religious belief case). Moreover, if the connection I drew between evidential incognizance and learned helplessness is well-made, there is reason to think that part of what is missing in cases of evidential incognizance is a sufficiently robust sense of one’s epistemic agency.

Isn’t a *good philosophical training* likely to remediate tendencies to over-generalize, to promote attention to sources of evidence we may overlook, and to promote a sense of epistemic agency, and thus to combat evidential incognizance at least in cases of these kinds? Philosophy seeks to understand the most fundamental and conceptually difficult questions in life, and thus is reluctant to view any matter as beyond the pale, or as beyond the ken of the inquiring mind. Many philosophical questions are evidently unanswerable by the methods of current natural science, and before encountering philosophy, many of us would have considered these questions unanswerable, or perhaps even foolish. In striving to answer such questions, philosophy develops new approaches and avenues of inquiry, and it sensitizes us to evidence and arguments that we would never have considered or thought relevant previously. Furthermore, the philosophical method teaches us to pay careful attention to details and distinctions, and to avoid careless over-generalizations. Finally, epistemology as a sub-field of philosophy develops our understanding of the distinction between what we know and what we don’t know, as well as of how knowledge is possible. Philosophical training promises thereby to expand the horizons of our sense of epistemic agency and to instill the kind of discerning attentiveness that runs counter to evidential incognizance.

While it is yet uncertain to what extent philosophical training could function as a general antidote for evidential incognizance, recognition of this epistemic vice and its causes helps us better understand how we sometimes fail to acquire knowledge. I hope it will also help us better understand the “new” propaganda and how to respond to it.

## Footnotes

1 I assume here a conception of evidence as facts, obvious examples being such facts as proton trails in cloud chambers, fingerprints, smoking guns, the existence of witness statements or news reports, and so on. While this is in line with a common-sense concept of evidence, philosophers have advocated alternative accounts of evidence, such as evidence as non-factive mental states (seemings), or evidence as the totality of what one knows. I cannot assess the arguments for such accounts here. Philosophers inclined toward them may translate my claims about sensitivity to what I call “evidence” into claims about sensitivity to epistemically relevant facts.

2 The understanding of epistemic vices here is loosely based on Cassam’s obstructivist definition (Cassam, 2019, p. 23).

3 I thank Nicholas Shackel for this point.

4 I thank Jason Kawall for the point that evidence need not necessarily be of interest.

5 I thank Heather Battaly for this suggestion.

6 I thank Bettina Bussmann for the idea to connect evidential incognizance to false balance in media.

## References

[CR1] Aristotle. (2009). *The Nicomachean ethics* (W. D. Ross & L. Brown, Trans.). Oxford University Press. (Original work published 350 B.C.E.).

[CR2] Batory, A., & Svensson, S. (2019). The use and abuse of participatory governance by populist governments. *Policy & Politics, 47*(2), 227–244. 10.1332/030557319X15487805848586

[CR3] Cassam, Q. (2019). *Vices of the mind: From the intellectual to the political* (1st ed.). Oxford University Press. 10.1093/oso/9780198826903.001.0001

[CR4] Clarke, J., & Newman, J. (2017). ‘People in this country have had enough of experts’: Brexit and the paradoxes of populism. *Critical Policy Studies, 11*(1), 101–116. 10.1080/19460171.2017.1282376

[CR5] Crisp, R. (2010). Virtue ethics and virtue epistemology. *Metaphilosophy, 41*(1–2), 22–40. 10.1111/j.1467-9973.2009.01621.x

[CR6] Dawson, A., & Innes, M. (2019). How Russia’s Internet Research Agency built its disinformation campaign. *The Political Quarterly, 90*(2), 245–256. 10.1111/1467-923X.12690

[CR7] Fricker, M. (2007). *Epistemic injustice: Power and the ethics of knowing*. Oxford University Press.

[CR8] HCLU. (2017). Short analysis of the proposed Hungarian Bill on foreign funded non-governmental organizations. https://hclu.hu/en/articles/short-analysis-of-the-proposed-hungarian-bill-on-funded-non-governmental-organizations-1

[CR9] Hookway, C. (2003a). Affective states and epistemic immediacy. *Metaphilosophy, 34*(1/2), 78–96.

[CR10] Hookway, C. (2003b). How to be a virtue epistemologist. In M. DePaul & L. Zagzebski (Eds.), *Intellectual virtue: Perspectives from ethics and epistemology* (pp. 183–202). Oxford University Press. 10.1093/acprof:oso/9780199252732.003.0009

[CR11] Hookway, C. (2006). Reasons for belief, reasoning, virtues. *Philosophical Studies, 130*(1), 47–70. 10.1007/s11098-005-3233-1

[CR12] Hopkins, V. (2023, August 25). *Yevgeny Prigozhin, renegade mercenary chief who rattled Kremlin*. The New York Times. https://www.nytimes.com/2023/08/25/world/europe/yevgeny-prigozhin-dead.html

[CR13] Kidd, I. J. (2019). Epistemic corruption and education. *Episteme, 16*(2), 220–235. 10.1017/epi.2018.3

[CR14] Kidd, I. J. (2024). Metaphilosophical myopia and the ideal of expansionist pluralism. *Journal of Philosophy of Education, 57*(4–5), 1025–1040. 10.1093/jopedu/qhad060

[CR15] King, N. L. (2021). *Excellent mind: Intellectual virtues for everyday life*. Oxford University Press.

[CR16] Maier, S. F., & Seligman, M. E. P. (2016). Learned helplessness at fifty: Insights from neuroscience. *Psychological Review, 123*(4), 349–367. 10.1037/rev0000033 27337390 10.1037/rev0000033 PMC4920136

[CR17] McDowell, J. (1996). *Mind and world: With a new introduction*. (1st Harvard University Press paperback ed) Harvard University Press.

[CR18] McNaughton, D. (1988). *Moral vision: An introduction to ethics*. Basil Blackwell.

[CR19] Medina, J. (2013). The epistemology of resistance: Gender and racial oppression, epistemic injustice, and resistant imaginations [electronic resource]. *Oxford University Press.*10.1093/acprof:oso/9780199929023.001.0001

[CR20] Mill, J. S. (1978). *On liberty* (8th ed.). Hackett Pub Co. (Original work published 1859).

[CR21] National Archives. (2019). Remarks by President Trump at turning point USA’s teen student action summit 2019 – The White House. https://trumpwhitehouse.archives.gov/briefings-statements/remarks-president-trump-turning-point-usas-teen-studentaction-summit-2019/

[CR22] Pomerantsev, P. (2014). *Nothing is true and everything is possible: The surreal heart of the new Russia* (1st ed.). Public Affairs.

[CR23] Pomerantsev, P. (2019). *This is not propaganda: Adventures in the war against reality*. Faber and Faber.

[CR24] RSF. (2023). The world press freedom index 2023. Reporters without borders. https://rsf.org/en/index

[CR25] Russell, A., & Schwartz, F. (2023, September 20). *‘Enfeebled’ UN fights for relevance in divided world*. Financial Times. https://www.ft.com/content/ffa99120-8551-4c8c-b351-1f974ba269e3

[CR26] Seligman, M. E., & Maier, S. F. (1967). Failure to escape traumatic shock. *Journal of Experimental Psychology, 74*(1), 1–9. 10.1037/h0024514 6032570 10.1037/h0024514

[CR27] Seskuria, N. (2021, September 13). *Why Putin is obsessed with ‘Foreign Agents’*. Foreign Policy. https://foreignpolicy.com/2021/09/13/vladimir-putin-foreign-agents-law-russia/

[CR28] Priebus, S., & Végh, Z. (2022). Hungary after the general elections. Down the road of autocratisation? *Südosteuropa Mitteilungen, 3*, 7–20.

